# Data Quality Improvement and Internal Data Audit of the Chinese Neonatal Network Data Collection System

**DOI:** 10.3389/fped.2021.711200

**Published:** 2021-10-04

**Authors:** Jianhua Sun, Yun Cao, Mingyan Hei, Huiqing Sun, Laishuan Wang, Wei Zhou, Xiafang Chen, Siyuan Jiang, Huayan Zhang, Xiaolu Ma, Hui Wu, Xiaoying Li, Yuan Shi, Xinyue Gu, Yanchen Wang, Tongling Yang, Yulan Lu, Wenhao Zhou, Chao Chen, Shoo K. Lee, Lizhong Du, The Chinese Neonatal Network, Shoo K. Lee

**Affiliations:** ^1^Department of Neonatology, Shanghai Children's Medical Center, School of Medicine, Shanghai Jiao Tong University, Shanghai, China; ^2^Division of Neonatology, Children's Hospital of Fudan University, Shanghai, China; ^3^Neonatal Center, Beijing Children's Hospital, Capital Medical University, Beijing, China; ^4^Division of Neonatology, Henan Children's Hospital, Children's Hospital Affiliated to Zhengzhou University, Zhengzhou, China; ^5^Division of Neonatology and Center for Newborn Care, Guangzhou Women and Children's Medical Center, Guangzhou, China; ^6^Department of Pediatrics, Children's Hospital of Philadelphia and University of Pennsylvania Perelman School of Medicine, Philadelphia, PA, United States; ^7^Division of Neonatology, The Children's Hospital of Zhejiang University School of Medicine, Hangzhou, China; ^8^Division of Neonatology, The First Bethune Hospital of Jilin University, Changchun, China; ^9^Division of Neonatology, Qilu Children's Hospital of Shandong University, Ji'nan, China; ^10^Division of Neonatology, Children's Hospital of Chongqing Medical University, Chongqing, China; ^11^NHC Key Laboratory of Neonatal Diseases (Fudan University), Children's Hospital of Fudan University, Shanghai, China; ^12^Center for Molecular Medicine, Pediatrics Research Institute, Children's Hospital of Fudan University, Shanghai, China; ^13^Maternal-Infant Care Research Centre, Mount Sinai Hospital, Toronto, ON, Canada; ^14^Department of Pediatrics, Mount Sinai Hospital, Toronto, ON, Canada; ^15^Department of Pediatrics, University of Toronto, Toronto, ON, Canada; ^16^Department of Obstetrics and Gynecology, University of Toronto, Toronto, ON, Canada; ^17^Dalla Lana School of Public Health, University of Toronto, Toronto, ON, Canada

**Keywords:** internal data audit, neonatal database, data quality improvement, neonatal network, preterm infant

## Abstract

**Background:** The Chinese Neonatal Network (CHNN) is a nationwide neonatal network that aims to improve clinical neonatal care quality and short- and long-term health outcomes of infants. This study aims to assess the quality of the Chinese Neonatal Network database by conducting an internal audit of data extraction.

**Methods:** A data audit was performed by independently replicating the data collection and entry process in all 58 tertiary neonatal intensive care units (NICU) participating in the CHNN. Eighty-eight data elements selected for re-abstraction were classified into three categories (critical, important, less important), and agreement rates for original and re-abstracted data were predefined. Three to five records were randomly selected at each site for re-abstraction, including one short- (0–7 days), two medium- (8–28 days), and two long-stay (more than 28 days) cases. Agreement rates for each data item were calculated for individual NICUs and across the network, respectively.

**Results:** A total of 283 cases and 24,904 data fields were re-abstracted. The agreement rates for original and re-abstracted data elements were 96.1% overall, and 97.2, 94.3, and 96.6% for critical, important, and less important data elements, respectively. Individual site variation for discrepancies ranged between 0.0 and 18.4% for all collected data elements.

**Conclusion:** The completeness, precision, and quality of data in the CHNN database are high, providing assurance for multipurpose use, including health service evaluation, quality improvement, clinical trials, and other research.

## Introduction

Preterm birth and low birth weight remain the single largest cause of neonatal mortality and morbidity among births worldwide (1). Optimizing neonatal outcomes in preterm newborn infants is a priority internationally (2). Consequently, many regional, national, and international neonatal networks have been established over the past two decades, with the aim of improving clinical neonatal quality of care (3). Standardized neonatal databases that collect neonatal clinical information are critical for transforming the raw data collected into evidence-based conclusions (4) through benchmarking, monitoring and feedback of neonatal outcomes, critical evaluation of clinical care practices and healthcare service delivery, and continuous quality improvement efforts. These databases may also provide robust platforms for neonatal research (5).

The quality of data is essential for good epidemiological study since poor data quality may lead to unreliable conclusions (6). There are several approaches to improve the precision and reliability of data abstracted from medical records including abstractor training, routine communication with abstractors, standardized application of electronic data entry, and data audit (7, 8). Data can be manually abstracted from patient medical records into a customized data entry application (8). The use of a clearly written data abstraction manual combined with training of abstractors may decrease the potential for abstractors to apply subjective judgment and impair accuracy of data abstraction (9, 10). A major limitation of data abstraction is transcription error due to manual data entry by abstractors (11). Computerized data entry can reduce transcription error since the application is always linked with the electronic database (8). Incorporation of data validation into the computer application can also increase the accuracy of data collection. Routine internal data audit is another efficient method for checking the precision and reliability of data abstraction, especially when data are abstracted by multiple abstractors at different sites, which may result in inter- or intrarater variability (12). During internal data audit, data are reabstracted by the same or different abstractors to determine the disagreement between the original abstraction and reabstraction which is a quantitative evaluation of data precision (10). Random sampling applied during the data audit can reduce the time, cost, and human labor required (12, 13).

The Chinese Neonatal Network (CHNN) was established in 2019, with the aim of creating a trustworthy national source of neonatal clinical information for research, benchmarking, quality improvement, and policy making. Consequently, quality assurance of the database is particularly important to make sure that the data collected are of high quality. The objective of this manuscript is to report the results of an internal audit of the CHNN database using a prospective cross-sectional study approach.

## Method

### CHNN Data Collection Systems

CHNN was established in 2019 and comprises 58 tertiary-level neonatal intensive care units (NICU) in 25 provinces across China. All participating NICUs are grade A level III NICUs authorized by the Health Administration of China. The sites were selected to provide a large cohort representative of the different geographic regions of China. Inclusion criteria for the CHNN database were:

1. Birth weight < 1,500 g or gestational age <32 weeks.
2. Neonates who received the treatment for at least 24 h.
3. Neonates who died in the NICU.

Exclusion criteria were:

1. Stillbirth.
2. Delivery-room death.
3. Infants transferred to non-participating hospitals within 24 h after birth.

Ethics approval was received from the Ethics Committee of the Children's Hospital of Fudan University (#CHFU 2018-296) and all participating NICUs for the development, compilation, data transfer, hosting, and analysis of the CHNN dataset. Only deidentified data are transferred to the data coordinating center. All data protocols and procedures comply with national, provincial, and local regulations for protecting patients' personal privacy and confidentiality.

A custom-built, stand-alone system based on MS Access was developed and used for data collection and transfer. Similar to the Canadian Neonatal Network, data are prospectively collected on maternal and neonatal demographics, antenatal and birth history, NICU admission, NICU treatment, health outcome, and hospital discharge (12). Trained abstractors in each hospital abstract data from patient medical records directly into dedicated computers using a standard abstractor manual of operations and procedures, with standardized definitions of variables.

**Figure 1:**
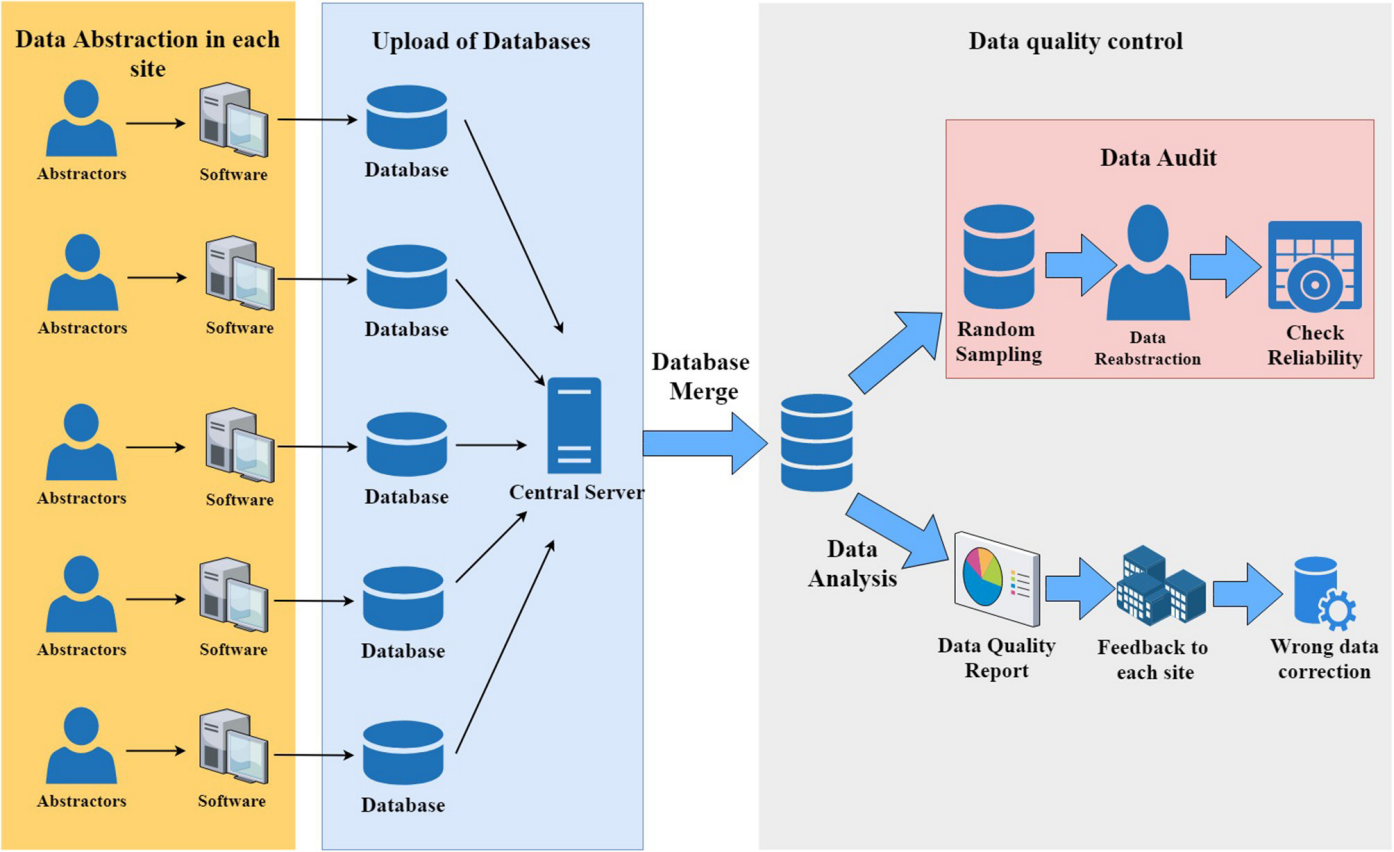
Data collection and data quality control in the Chinese Neonatal Network data collection system.

Each participant hospital has at least one dedicated abstractor, who is supervised by hospital site investigators and a single coordinator at the CHNN Coordinating Center, who answers questions about the process of data entry. Automated validation checks are incorporated in the data-entry software, and patient identifiers are stripped before data are submitted to the coordinating center. The central coordinator also checks the database regularly and writes the report for data quality. After submission, data are automatically checked by an error checking program and potential errors are fed back to each site for data recheck and correction (Figure 1). A randomized data audit was conducted after the data correction process to assess the quality of the data.

Data collection in the CHNN database started from Jan. 1st, 2019. The audited data was obtained from newborns who were admitted to CHNN NICUs between Jan 1, 2019 and Nov 31, 2019. During this time, there were 8,103 validated cases in the CHNN database.

### Sampling

Three to five cases were randomly selected in each hospital based on the number of NICU admissions, i.e., three cases for smaller hospitals and five cases for larger hospitals. Within each hospital, stratified random sampling was based on duration of hospitalization in three categories—short (0–7 days), medium (8–28 days), and long (>28 days) stay. The number of cases selected for audit in each category was prorated according to the distribution of cases in each category among the 58 CHNN sites. Accordingly, 53 short-stay, 108 medium-stay, and 122 long-stay cases were selected, for a total sample size of 283 cases for the data audit.

### Variable Selection for Data Reabstraction

We used the method of Shah et al. (12) to divide all data elements, including demographic, procedural, diagnostic, and outcome information, into three categories (critical, important, less important) based on the ideal level of required reliability of each item. The expected agreement rate of the three categories were ≥95, ≥90, and ≥85% concordance for critical, important, and less-important elements, respectively, which is the tolerable criteria for the data abstraction. Thirty elements were categorized as critical, 28 elements as important, and 30 elements as less important. The criteria for inclusion of these variables in each category were based on the need to test the precision of target variable and the possibilities of occurrence of subjective judgment. If the data elements did not meet the expected agreement rate, the coordinating center will analyze the potential problems in the process of data collection by communication with the site abstractor and investigators, and check the definition, structure, and purpose of variable itself. Furthermore, we also checked to ensure that selected variables were representative of all 360 variables in the entire CHNN databases by calculating the total agreement rate for 360 variables.

### Distribution of Sampling Results

After selection of variables, the coordinating center used the sampling criteria to randomly select cases from the original database for reabstraction and informed the sites accordingly. Within the following month, abstractors used a separate data reabstraction software to reenter data for cases randomly selected for data reabstraction. Abstractors were not permitted to revisit and review the original abstraction for checking of accuracy of data entry. The average time required for reabstraction of the 88 variables required for the audit was 50 min. This contrasts with the average of 60 min required for the 360 variables that are routinely abstracted for a typical patient and indicates the complexity of the variables chosen for reabstraction. Consequently, the total extra workload averaged between 2 and 3 h for each participating CHNN hospital. All CHNN hospitals participated in the data audit.

### Data Analysis

Data analysis and random sampling were conducted using SAS version 9.4. Before data analysis, records of selected cases were extracted from the original CHNN database and categorized into the three abovementioned categories. The total agreement rates of each variable in these three categories were analyzed and evaluated using the following formula:


$$\text{Agreement rate}=\frac{\text{Number of agreement between all original and reabstracted case}}{\text{total number of selected cases }}$$

For each participant site, the range of agreement rate was reported, which reflects the percentage of reabstracted record that agreed with original abstraction in each hospital of CHNN. Descriptive analysis was applied to report the median gestational age and length of stay of cases.

### Pilot Study

A pilot study was conducted at the Children's Hospital of Fudan University 3 months prior to the CHNN audit exercise to test the data reabstraction software and ensure that the audit exercise could be carried out smoothly. We sampled three cases at random for the pilot study. Data analysis of the pilot study confirmed that the process could be executed efficiently.

### Ethics

Ethics approval was obtained from the Children's Hospital of Fudan University to collect, store, and analyze the data from newborn infants admitted to all participant sites of CHNN. Ethics approvals were also obtained from all participating CHNN sites for local data collection, storage, and transfer of patient data to the CHNN Coordinating Center for analysis. All necessary health information privacy and security procedures were followed and conformed with local, provincial, and national requirements and guidelines. Prior to data transfer to the Coordinating Center, all patient identifiers were stripped to ensure privacy.

## Results

During the study period, all 58 CHNN sites participated in the audit. A total of 283 cases were reabstracted. Among them, 53 cases had short duration of hospitalization (<8 days), 108 cases had medium duration of hospitalization (8–28 days), and 122 cases had long duration of hospitalization (>28 days). Twenty-nine cases are abstracted by different abstractors (10.25%) and 254 cases are abstracted by the same abstractors (89.75%) of the original chart abstraction. The median gestational age of audited cases was 34 weeks (range, 22–41 weeks). The median length of stay of audited cases was 10 days (range, 4–21 days), which were reflective of the overall CHNN population ranges. The agreement rate was 96.1% overall for all 88 selected data elements, 97.2% for critical data elements, 94.3% for important data elements, and 96.7% for less important data elements (Table 1). When segregated by length of hospital stay, the agreement rate was 95.8% for short-stay variables, 96.1% for medium-stay variables, and 96.3% for long-stay variables. Individual site variation for agreement ranged between 81.6 and 100% for all collected data elements.

**Table 1:** Overall agreement rate for all selected audit cases.

**Data elements**	**Total agreement**	**Agreement for short stay**	**Agreement for medium stay**	**Agreement for long stay**	**Site range of agreement rate%**
Critical	8,255 (97.2%)	1,547 (97.3%)	3,265 (97.2%)	3,443 (97.3%)	83.3–100.0
Important	7,472 (94.3%)	1,399 (94.3%)	2,951 (94.1%)	3,122 (94.5%)	78.6–100.0
Less important	8,207 (96.7%)	1,521 (95.7%)	3,251 (96.8%)	3,435 (97.0%)	82.7–100.0
Total	23,934 (96.1%)	4,467 (95.8%)	9,467 (96.1%)	10,000 (96.3%)	81.6–100.0

For critical data elements, the individual site agreement rate ranged from 83.3 to 100.0% (Table 1). For individual critical data elements, the agreement rate ranged from 100% for admission status, indomethacin for patent ductus arteriosus, ligation of ductus arteriosus, and postnatal steroids to 90.46% (range, 20–100%) for neurological findings (Table 2). Data elements that exceeded the pre-established <5% discrepancy threshold were receipt of antenatal corticosteroids, patent ductus arteriosus diagnosed or treated, and retinopathy of prematurity. The disagreement rate for antenatal corticosteroids was twice as high at Children's Hospitals (10 cases) compared with Maternity Hospitals (five cases) due to poor information transfer from the birth hospitals. There were 11 cases where misclassification for the question “Was screening for ROP performed or not?” led to errors in ROP-related variables.

**Table 2:** Agreement number and percentage for critical variables.

**Data element**	**Agreement *n* (%)**	**Range of agreement rate %**
Admission information	269 (95.1%)	60.0–100.0
Maternal/obstetric risks and treatments	263 (92.9%)	0–100.0
Positive blood or cerebrospinal fluid cultures	279 (98.6%)	60.0–100.0
Patent ductus arteriosus diagnosed	262 (92.6%)	40.0–100.0
Patent ductus arteriosus treated	267 (94.4%)	40.0–100.0
Necrotizing enterocolitis	280 (98.9%)	80.0–100.0
Stage of retinopathy of prematurity	261 (92.2%)	60.0–100.0
Chest compression at birth	279 (98.6%)	80.0–100.0
Intraventricular hemorrhage and stages	252 (89.1%)	20.0–100.0
Surfactant type	273 (96.5%)	60.0–100.0
Type of postnatal steroids	279 (98.6%)	80.0–100.0
Discharge destination	281 (99.3%)	80.0–100.0
Discharge date	280 (98.9%)	80.0–100.0

For important data elements, the individual site agreement rate ranged from 78.6 to 100% (Table 1). For individual important data elements, the agreement rate ranged from 100% for laparotomy, ventriculoperitoneal shunt, and exchange transfusion to 85.9% (range 0–100%) for suspected chorioamnionitis (Table 3). Data elements which exceeded the threshold discordance rate of 10% were course and timing of antenatal corticosteroids, receipt of more than one course of antenatal corticosteroids, and suspected chorioamnionitis. The discrepancy was higher among outborn compared with inborn cases, for receipt of more than one course of AC (17 outborn vs. 12 inborn), timing of AC, and suspected chorioamnionitis (23 outborn vs. 17 inborn).

**Table 3:** Agreement number and percentage for important variables.

**Data element**	**Agreement *n* (%)**	**Range of agreement rate %**
Admission		
Apgar score at 1 min	278 (98.2%)	80.0–100.0
Apgar score at 5 min	277 (97.9%)	75.0–100.0
Maternal/obstetric		
If received antenatal corticosteroids, course and timing	250 (88.3%)	40.0–100.0
Receipt of more than one course of antenatal corticosteroids	254 (89.6%)	20.0–100.0
Total courses of corticosteroids given	273 (96.5%)	60.0–100.0
Rupture of membranes	259 (91.5%)	40.0–100.0
Maternal diabetes	272 (96.1%)	60.0–100.0
Received magnesium sulfate	265 (93.6%)	40.0–100.0
Suspected chorioamnionitis	243 (85.9%)	0.0–100.0
Maternal age	264 (93.3%)	40.0–100.0
Receipt of prenatal care	270 (95.4%)	40.0–100.0
Diagnoses/procedures		
Laparotomy	283 (100.0%)	100.0–100.0
Ventriculoperitoneal shunt	283 (100.0%)	100.0–100.0
Thoracotomy	282 (99.7%)	80.0–100.0
Exchange transfusion	283 (100.0%)	100.0–100.0
Intestinal perforation	279 (98.6%)	80.0–100.0
Retinopathy screening	272 (96.1%)	60.0–100.0
Plus disease in left eye	256 (90.5%)	40.0–100.0
Plus disease in right eye	257 (90.8%)	40.0–100.0
Congenital anomalies	263 (92.9%)	40.0–100.0
Diagnosis of infection	256 (90.5%)	20.0–100.0
Neurological findings		
Neuroimaging results	257 (90.8%)	40.0–100.0
Periventricular echogenicity on left side	270 (95.4%)	40.0–100.0
Intraprenchymal hemorrhage on right side	268 (94.7%)	20.0–100.0
Transport Risk Index of Physiologic Stability (TRIPS)		
Admission blood pressure	261 (92.2%)	20.0–100.0
Temperature	262 (92.6%)	20.0–100.0
Discharge		
Oxygen percentage at discharge	270 (95.4%)	40.0–100.0
Discharge against medical advice (DAMA)	265 (93.6%)	60.0–100.0

For less-important data elements, the individual site agreement rate ranged from 82.7 to 100.0% (Table 1). For individual less-important data elements, the agreement rate ranged from 100% for procedures like ostomy, necrotizing enterocolitis peritoneal drainage, gastrostomy, and tracheostomy to 87.6% (range, 20.0–100.0%) for stimulus response appropriate for GA (Table 4). None of the data elements in this category crossed the threshold discordance rate of 15%.

**Table 4:** Agreement number and percentage for less-important variables.

**Data element**	**Agreement *n* (%)**	**Range of agreement rate %**
Admission		
Documented gestational age (day)	279 (98.6%)	80.0–100.0
Maternal/obstetric		
Gravida	279 (98.6%)	80.0–100.0
Parity	263 (92.9%)	50.0–100.0
Total abortions	275 (97.2%)	80.0–100.0
Antenatal intervention	265 (93.6%)	20.0–100.0
Number of births	273 (96.5%)	60.0–100.0
Cultures/transfusions		
Platelet transfusions	281 (99.3%)	80.0–100.0
Immunoglobulin	281 (99.3%)	80.0–100.0
Diagnoses/procedures		
Respiratory distress syndrome	265 (93.6%)	60.0–100.0
Ostomy	283 (100.0%)	100.0–100.0
Necrotizing enterocolitis peritoneal drainage	283 (100.0%)	100.0–100.0
Retinopathy zone on left side	268 (94.7%)	60.0–100.0
Retinopathy zone on right side	269 (95.1%)	60.0–100.0
No active resuscitation needed	266 (94.0%)	40.0–100.0
Positive pressure ventilation via bag and mask	266 (94.0%)	40.0–100.0
Positive pressure ventilation via endotracheal tube	269 (95.1%)	60.0–100.0
Epinephrine	281 (99.3%)	60.0–100.0
Other resuscitation	283 (100.0%)	100.0–100.0
Transport Risk Index of Physiologic Stability (TRIPS)		
Respiratory status	252 (89.1%)	40.0–100.0
Stimulus response appropriate for GA	248 (87.6%)	20.0–100.0
Transport		
Pickup date	268 (94.7%)	40.0–100.0
Arrival date	268 (94.7%)	40.0–100.0
Medications		
Postnatal steroids indication	277 (97.9%)	80.0–100.0
Postnatal steroids route	278 (98.2%)	80.0–100.0
Discharge		
Ostomy	283 (100.0%)	100.0–100.0
Gastrostomy	283 (100.0%)	100.0–100.0
Ventilation	281 (99.3%)	80.0–100.0
Continuous positive airway pressure	282 (99.7%)	80.0–100.0
Tracheostomy	283 (100.0%)	100.0–100.0
Discharge weight	275 (97.2%)	20.0–100.0

The overall agreement of all 360 data variables in the entire database was 96.3% for the *n* = 98,100 variables. The agreement rate for cases with short duration of hospitalization was 96.0% (*n* = 20,144 variables), medium duration of hospitalization was 95.9% (*n* = 42,549 variables), and long duration of hospitalization was 96.1% (*n* = 44,890 variables).

## Discussion

To our knowledge, this is the first study of data quality in a neonatal database in China. This internal audit with participation of all CHNN sites showed a high rate of agreement between the reabstracted and original data collected for the CHNN database, which is the largest neonatal database of its kind in China, with participation of 58 NICUs in 25 provinces throughout China. The overall agreement rate was high at 96.1%, and the disagreement rates of 2.8% for critical variables, 5.7% for important variables, and 3.3% for less-important variables, met their prescribed targets of <5, <10, and <15%, respectively. There was low site-to-site variation in the disagreement rate, ranging from perfect agreement to 18.4% disagreement for all collected data elements. These results demonstrate that the CHNN data have high precision and reliability and is suitable for multiple uses, including research.

Our results are comparable with audits performed by large neonatal networks in other countries. The Canadian Neonatal Network reported a high precision of data quality collection (agreement rate of 96.9% overall, 98% for critical, 96.1% for important, and 96.3% for less-important data elements) with small individual site variation for discrepancies (0.2–12.8%) through a similar internal audit process (12). The Vermont-Oxford Trials Network conducted a similar evaluation from 635 of 4,341 eligible infants across 40 sites by reviewing 10 critical data elements of their medical record. The disagreement rate ranged from 1.3% for birth date to 8.8% for discharge date, and 90% of disagreements were due to errors in transcription or interpretation, rather than data keying errors (14). An assessment of the neonatal database maintained by the United Kingdom National Neonatal Research Database revealed low discordance rate of <5% for patient characteristics, treatment, and clinical outcomes (5).

We found that disagreement between the reabstracted and original data were mainly due to missing maternal data and incomplete records in the patient medical charts rather than poor abstraction of data. Missing data secondary to poor patient medical chart documentation by healthcare providers is a documented phenomenon (15). In our study, many outborn infants in Children's Hospitals in CHNN lack maternal information such as the use of AC (including timing and course) and chorioamnionitis, because maternal information is often not routinely provided when the infant is transferred from the maternity hospital. Although statistical methods such as multiple imputations can be applied when maternal data is missing, these methods have their own limitations. Another common reason for disagreement between the reabstracted and original data was the ambiguity of some information recorded in the electronic medical record (EMR). For example, in some CHNN sites, abstractors had difficulty distinguishing suspected from histologically diagnosed chorioamnionitis because only chorioamnionitis was recorded in the EMR. Misinterpretation of the definition of chorioamnionitis by some abstractors added to the confusion and further compounded the error. Although this was the first year of data collection in CHNN, we did not find significant data keying errors. Horbar et al. reported that data keying errors in the Vermont-Oxford Trials Network decreased from 13.7 to 3.7% over time with improvements in data keying procedures (14). This is encouraging, and we anticipate that CHNN data quality will further improve as our procedures are streamlined over time.

The high precision and reliability of the database is due at least in part to the multiple layers of quality assurance built into the data collection system (16). These include central coordination, dedicated data entry personnel with uniform training by the coordinating center, a single manual of operations and protocols with standardized definitions, direct data entry into a computer, use of a unique data entry program with built-in error checking, multiple levels of error checking after data entry, feedback of errors to participating sites with a protocol for correcting errors, and annual audit of the data collected. In this regard, this audit was also useful because it highlighted data problems that were not previously apparent and CHNN has since initiated a network-wide effort to resolve the difficulties identified, such as missing maternal information and ambiguity of medical chart records. Consequently, we anticipate that the precision of the database will further improve in the future.

There are several limitations to this internal audit. Our audit exercise involved only a small random sample and was only conducted once per year. However, to reabstract and audit all cases in the database would be prohibitively time consuming, expensive, and impractical. Consequently, most databases only conduct small random sampling audits similar to our study (12, 17). An external audit using different data abstractors is considered more reliable than an internal audit. However, it is labor intensive, costly, and time consuming because external abstractors need to deal with unfamiliar medical record systems and protocols, and they collect data retrospectively rather than prospectively. Consequently, external audits are seldom employed in audits of network databases (12).

In conclusion, our audit demonstrated that data from the CHNN data collection system show high precision and reliability. With the implementation of measures designed to improve data quality based on deficiencies identified in this audit, we expect that data quality will further improve. As our network matures, periodic data audit will be essential to ensure the reliability of our database for research, and funding, manpower, and resources will be needed to support the practice of data quality improvement.

## Data Availability Statement

The original contributions presented in the study are included in the article/Supplementary Material, further inquiries can be directed to the corresponding authors.

## Ethics Statement

The Ethics Committee from the Children's Hospital of Fudan University has approved CHNN to collect, store and analyse the overall data from all participant sites of CHNN. For individual sites, there is also approval from the ethics committee for local data collection and storage. Health information for each case will be well protected and maintained at local sites with a unique ID, which will not be submitted in the coordinate center. Since audit practice is the part of the current exercise of the project, specific approval will not be warranted.

## Author Contributions

JS, SL, and LD: study concept and design. JS, XC, SL, and LD: acquisition, analysis, or interpretation of data. JS and XC: drafting of the manuscript. XC, YW, and XG: statistical analysis. TY and YL: administrative, technical, or material support. JS, WenZ, CC, SL, and LD: study supervision. All authors are critical revision of the manuscript for important intellectual content. All authors contributed to the article and approved the submitted version.

## Group Information of the Chinese Neonatal Network

Chairmen: Shoo K. Lee, MBBS, Mount Sinai Hospital, University of Toronto; Chao Chen, MD, Children's Hospital of Fudan University. Vice-Chairmen: Lizhong Du, MD, Children's Hospital of Zhejiang University School of Medicine; Wenhao Zhou, Children's Hospital of Fudan University. Site principle investigators of the Chinese Neonatal Network: Children's Hospital of Fudan University: Yun Cao, MD; The Third Affiliated Hospital of Zhengzhou University: Falin Xu, MD; Tianjin Central Hospital of Obstetrics and Gynecology: Xiuying Tian, MD; Guangzhou Women and Children's Medical Center: Huayan Zhang, MD; Children's Hospital of Shanxi: Yong Ji, MD; Northwest Women's and Children's Hospital: Zhankui Li, MD; Gansu Provincial Maternity and Child Care Hospital: Jingyun Shi, MD; Shengjing Hospital of China Medical University: Xindong Xue, MD; Shenzhen Maternity and Child Health Care Hospital: Chuanzhong Yang, MD; Quanzhou Women and Children's Hospital: Dongmei Chen, MD; The Affiliated Suzhou Hospital of Nanjing Medical University: Sannan Wang, MD; Guizhou Women and Children's Hospital/Guiyang Children's Hospital: Ling Liu, MD; Hunan Children's Hospital: Xirong Gao, MD; The First Bethune Hospital of Jilin University: Hui Wu, MD; Fujian Maternity and Child Health Hospital, Affiliated Hospital of Fujian Medical University: Changyi Yang, MD; Nanjing Maternity and Child Health Care Hospital: Shuping Han, MD; Qingdao Women and Children's Hospital: Ruobing Shan, MD; The Affiliated Hospital of Qingdao University: Hong Jiang, MD; Children's Hospital of Shanghai: Gang Qiu, MD; Women and Children's Hospital of Guangxi Zhuang Autonomous Region: Qiufen Wei, MD; Children's Hospital of Nanjing Medical University: Rui Cheng, MD; Henan Children's Hospital: Wenqing Kang, MD; The First Affiliated Hospital of Xinjiang Medical University: Mingxia Li, MD; Foshan Women and Children's Hospital: Yiheng Dai, MD; The First Affiliated Hospital of Anhui Medical University: Lili Wang, MD; Shanghai First Maternity and Infant Hospital: Jiangqin Liu MD; Yuying Children's Hospital Affiliated to Wenzhou Medical University: Zhenlang Lin, MD; Children's Hospital of Chongqing Medical University: Yuan Shi, MD; The First Affiliated Hospital of Zhengzhou University: Xiuyong Cheng, MD; The First Affiliated Hospital of USTC, Division of Life Sciences and Medicine, University of Science and Technology of China: Jiahua Pan, MD; Shaanxi Provincial People's Hospital: Qin Zhang, MD; Children's Hospital of Soochow University: Xing Feng, MD; Wuxi Maternity and Child Healthcare Hospital: Qin Zhou, MD; People's Hospital of Xinjiang Uygur Autonomous Region: Long Li, MD; The Second Xiangya Hospital of Central South University: Pingyang Chen, MD; Qilu Children's Hospital of Shandong University: Xiaoying Li, MD; Hainan Women and Children's Hospital: Ling Yang, MD; Xiamen Children's Hospital: Deyi Zhuang, MD; Xinhua Hospital affiliated to Shanghai Jiao Tong University School of Medicine: Yongjun Zhang, MD; Shanghai Children's Medical Center, School of Medicine, Shanghai Jiao Tong University: Jianhua Sun, MD; Shenzhen Children's Hospital: Jinxing Feng, MD; Children's Hospital Affiliated to Capital Institute of Pediatrics: Li Li, MD; Women and Children's Hospital, School of Medicine, Xiamen university: Xinzhu Lin, MD; General Hospital of Ningxia Medical University: Yinping Qiu, MD; First Affiliated Hospital of Kunming Medical University: Kun Liang, MD; Hebei Provincial Children's Hospital: Li Ma, MD; Jiangxi Provincial Children's Hospital: Liping Chen, MD; Fuzhou Children's Hospital of Fujian Province: Liyan Zhang, MD; First Affiliated Hospital of Xian Jiao Tong University: Hongxia Song, MD; Dehong people's Hospital of Yunnan Province: Zhaoqing Yin, MD; Beijing Children's Hospital, Capital Medical University: Mingyan Hei, MD; Zhuhai Center for Maternal and Child Health Care: Huiwen Huang, MD; Guangdong Women and Children's Hospital: Jie Yang, MD; Dalian Municipal Women and Children's Medical Center: Dong Li, MD; Peking Union Medical College Hospital: Guofang Ding, MD; Obstetrics & Gynecology Hospital of Fudan University: Jimei Wang, MD; Shenzhen Hospital of Hongkong University: Qianshen Zhang, MD; Children's Hospital of Zhejiang University School of Medicine: Xiaolu Ma, MD.

## Funding

This work was funded by the Canadian Institutes of Health Research [CTP87518 to SL].

## Conflict of Interest

The authors declare that the research was conducted in the absence of any commercial or financial relationships that could be construed as a potential conflict of interest.

## Publisher's Note

All claims expressed in this article are solely those of the authors and do not necessarily represent those of their affiliated organizations, or those of the publisher, the editors and the reviewers. Any product that may be evaluated in this article, or claim that may be made by its manufacturer, is not guaranteed or endorsed by the publisher.
